# MEMS Energy Harvesting: Enabling Self-Powered Solutions

**DOI:** 10.3390/mi17070816

**Published:** 2026-07-07

**Authors:** Yu Jia

**Affiliations:** School of Engineering and Innovation, Aston University, Birmingham B4 7ET, UK; y.jia1@aston.ac.uk

Energy harvesting is a broad family of diverse technologies that scavenge various types of ambient energy (such as light, vibration, human motion, thermal, radiofrequency (RF), radioisotope, etc.) to provide self-sustaining power to microelectronics and wireless sensors in a decentralised manner [1]. A key rationale for energy harvesting is to either replace or complement batteries to avoid the costly endeavour of battery replacements.

This is particularly relevant in applications where devices are remote, buried or in hard-to-access locations. With the increasing number of Internet-of-Things (IoT) wireless nodes, labour cost to service batteries can also become prohibitive. Energy harvesting, on the other hand, holds the promise to enable “fit-and-forget” solutions [1].

For applications where device size is not a constraint [2], such as condition monitoring of large civil infrastructural systems such as bridges or tunnels, macro-scaled harvesters are typically preferred to maximise power output. At the microscale [3], micro-electromechanical systems (MEMS) and film-based energy harvesters have been proposed as on-board or on-chip solutions to self-power other micro-devices such as wireless sensors or medical implants. Furthermore, batteries have increasingly become a sticking point in the ever-evolving arena of device miniaturisation. MEMS energy harvesters’ compatibility with semiconductor fabrication and packaging processes holds the promise to potentially minimise the physical footprint of the on-board power supply.

It has been approximately a quarter of a century since some of the early contemporary research on MEMS energy harvesting was initiated [4,5]. The field has witnessed a plethora of publications ranging from piezoelectric energy harvesters [6] to triboelectric nanogenerators [7], from control and tuning mechanisms [8] to passive broadband designs [9], and from various nonlinear dynamics phenomena [10] to different power conditioning strategies [11]. Yet, the bulky battery remains unmoved as the dominant power source for wireless microsystems in most practical deployments. Multiple commercialisation ventures such as MicroGen’s MEMS vibration energy harvester [12], TEGMAT’s thermal energy harvester [13], e-peas’ energy harvesting power management chip [14], and many more, have yet to shift the paradigm.

This observation is not a critique of the research and commercialisation efforts, to date; it reflects the scale of the challenge that needs to be overcome. MEMS energy harvesting operates at scales where inertial mass (and thus power) is small, damping is relatively large, and available ambient energy is diffuse and spectrally complex. The typical power output of an optimised MEMS energy harvester is measured in the order of microwatts to low milliwatts [15]. While this power regime does align with ultra-low-power wireless sensors and microcontrollers [15], there is little margin for inefficiency or redundancy, or for accommodating complex tasks or handling large volumes of data.

Much of the early research in the first decade of this century established the analytical framework and demonstrated proof-of-concept prototypes harvesting energy via various transduction mechanisms [1,15]. Throughout the second decade, there were diverse efforts to improve power density, broaden the operational bandwidth, enhance power conditioning efficiency, and explore new higher power transducer materials [3,10]. More recently, energy harvesting is rarely seen as a standalone technology, but increasingly integrated into the wider microsystems’ ecosystem, with ongoing pursuits to bridge the power gap between supply and demand [7].

This evolution over time has also been accompanied by increasing diversification of transduction mechanisms for a wide range of applications. Table 1 illustrates the breadth and diversity of transduction mechanisms that have been explored for MEMS energy harvesting, reflecting the community’s sustained effort to match device physics to the specific energy sources available in target deployment environments. However, these mechanisms are not equally competitive across all application contexts.

Among vibration-based mechanisms, piezoelectric and triboelectric have risen from the pack to become the most promising contenders due to the potential for high power density. While lead zirconate titanate (PZT) was traditionally the dominant choice of piezoelectric material due to its high charge constant, its journey towards microfabrication has been a rocky one, yielding mixed results at best. Coupled with the wider trend of moving away from lead-based materials, the field has increasingly explored MEMS compatible and non-lead alternatives, such as aluminium nitride (AlN), zinc oxide (ZnO), barium titanate (BTO), potassium sodium niobate (KNN), etc.

Resonance optimisation has always plagued vibration-based energy harvesting, with broadband and nonlinear strategies [10] yielding mixed results, to date. Triboelectric nanogenerators sit in a unique position within the kinetic energy harvesting family, operating effectively at low frequencies and inconsistent motion. The wide range of materials available for triboelectric harvesters also offers potential for microfabrication integration.

Photoelectric and thermoelectric generators are both IC-compatible solid-state solutions with no moving parts. This potentially improves robustness and minimises packaging complexity associated with dynamic MEMS. Photoelectric transducers also offer one of the highest power densities when harvesting from direct sunlight. However, power density drops by at least an order of magnitude for indoor lighting and is entirely unsuitable for enclosed environments with no external light source. Thermoelectric is suitable for environments where a sustained temperature gradient can be found, yielding a reliable and continuous power output. However, the thermoelectric efficiency benefits from a large gradient, which can be non-trivial to maintain (more than 1–5 °C) within the small physical footprint of a microsystem solution [15].

Furthermore, there have also been many other niche research efforts into harvesting ambient RF energy, biochemical energy for implants, and radioisotope energy in entirely isolated and enclosed environments; each with their own compelling use case. There is no one-size-fit-all solution, or a “super harvester”, capable of dominating all application scenarios. The ultimate question is not which transducer, design, or mechanism produces the highest peak power density under idealised laboratory conditions, but which is the most suitable for a specific application context based on environmental constraints, reliability, and load requirements. Multi-source and hybrid solutions can also complement each other to capture a wider spectrum.

With the parallel rise of IC-compatible solid-state batteries, the perceived rivalry between batteries and energy harvesting has started to evolve into more of a complementary symbiosis. As power conditioning efficiency continues to improve, transducer power density continues to rise, and ultra-low-power electronics continue to be more efficient; the available power budget produced from energy harvesting starts to become less scarce.

While the exaggerated over-hype and over-promise during nascent stages, such as Nokia’s claims of developing a self-charging phone [16], may have resulted in disappointment and reputational cost; the energy harvesting research field has since matured into a well-established community that is tightly integrated with wider microsystems, functional materials and wireless sensing communities. There might not be a revolutionary step change or a tsunami of adoption awaiting energy harvesting, but its thesis of enabling fit-and-forget solutions remains fundamentally solid. As more of the persisting technical challenges are overcome, we may witness the silent rise of energy harvesting as a decentralised energy backbone across many of the other emerging technologies.

## Figures and Tables

**Table 1:** Major types of transducers employed for MEMS energy harvesting ^1^ [1].

Transducer	Typical Power	Comments
Piezoelectric	1’s µW to 1’s mW	High power density, scalable, limited by narrow bandwidth, IC compatible for some materials.
Electromagnetic	1’s µW to 100’s µW	Modest power density, limited by narrow bandwidth, not entirely IC compatible, not scalable.
Electrostatic	0.1’s µW to 100’s µW	Low power density, fully IC compatible.
Triboelectric	10’s µW to 1’s mW	High power density, suitable for low frequency, partially IC compatible, scalable, limited by wear and tear and impedance matching challenges.
Magnetostrictive	1’s µW to 100’s µW	Modest to low power density, sensitive to strain and magnetic perturbation, not IC compatible.
Thermoelectric	1’s µW to 100’s µW	Moderate power density, scalable, solid-state, IC compatible, ideal for high temperature gradients.
Pyroelectric	0.1’s µW to 10’s µW	Low power density, needs temperature fluctuation, IC compatible, shares piezoelectricity.
Photoelectric	10’s µW to 1’s mW	Sunlight is better than indoor light for power density, scalable, IC compatible, solid state.
Radiofrequency	0.1’s µW to 1’s µW	Low power density, generally consistent source, IC compatible, solid state.
Radioisotope	0.1’s µW to 10’s µW	Low power density, consistent source, not entirely IC compatible, regulatory constraints.
Biochemical	1’s µW to 10’s µW	Glucose or other biochemical fuel source, low power density, suitable for implantables.

^1^ Assuming scale constrained within 1 cm^3^ for dynamic transducers or 1 cm^2^ for surface area-based solid-state transducers. Typical power values are indicative order-of-magnitude estimates.

## Abbreviations

The following abbreviations are used in this manuscript:

RF	Radiofrequency
MEMS	Micro-Electromechanical System
IoT	Internet-of-Things
IC	Integrated Circuit
PZT	Lead Zirconate Titanate
AlN	Aluminium Nitride
BTO	Barium Titanate
KNN	Potassium Sodium Niobate

## References

[1] Priya S., Inman D.J. (2009). Energy Harvesting Technologies.

[2] Jia Y., Yan J., Du S., Feng T., Fidler P., Middleton C., Soga K., Seshia A.A. (2017). Real world assessment of an auto-parametric electromagnetic vibration energy harvester. J. Intell. Mater. Syst. Struct..

[3] Selvan K.V., Ali M.S.M. (2016). Micro-scale energy harvesting devices: Review of methodological performances in the last decade. Renew. Sustain. Energy Rev..

[4] Ramsay M.J., Clark W.W. Piezoelectric energy harvesting for bio-MEMS applications. Proceedings of the SPIE Smart Structures and Materials + Nondestructive Evaluation.

[5] Roundy S., Wright P.K. (2004). A piezoelectric vibration based generator for wireless electronics. Smart Mater. Struct..

[6] Anang F.E.B., Wei X., Xu J., Cain M., Li Z., Brand U., Peiner E. (2024). Area-Selective Growth of Zinc Oxide Nanowire Arrays for Piezoelectric Energy Harvesting. Micromachines.

[7] Wu L., Ren Z., Wang Y., Tang Y., Wang Z.L., Yang R. (2024). Miniaturized and High Volumetric Energy Density Power Supply Device Based on a Broad-Frequency Vibration Driven Triboelectric Nanogenerator. Micromachines.

[8] Wang M., Jiang Z., Jiang W., Feng X., Ding J., Sun Y., Pu H., Liao S. (2025). Active–Passive Vibration Control of Cantilever Beam Based on Magnetic Spring with Negative Stiffness and Piezoelectric Actuator. Micromachines.

[9] Ahmed M.S., Ma X., Jia Y. (2026). Dual-Mode, Orientation-Adaptive Broadband Rotational Energy Harvester for Diverse Noise and Vibration Environments. Micromachines.

[10] Jia Y. (2020). Review of nonlinear vibration energy harvesting: Duffing, bistability, parametric, stochastic and others. J. Intell. Mater. Syst. Struct..

[11] Szarka G.D., Stark B.H., Burrow S.G. (2011). Review of Power Conditioning for Kinetic Energy Harvesting Systems. IEEE Trans. Power Electron..

[12] MicroGen’s Piezo-MEMS Vibration Energy Harvesters. https://www.analog.com/jp/resources/technical-articles/microgen-s-piezo-mems-vibration-energy-harvesters-enable-linear-technology-smartmesh-ip-wireless.html.

[13] Thermal Energy Harvesting. https://www.tegmat.com/.

[14] E-Peas: The Most Efficient Energy Harvesting PMICs. https://e-peas.com/.

[15] Mitcheson P.D., Yeatman E.M., Rao G.K., Holmes A.S., Green T.C. (2008). Energy harvesting from human and machine motion for wireless electronic devices. Proc. IEEE.

[16] Nokia Developing Phone that Recharges Itself Without Mains Electricity. https://www.theguardian.com/environment/2009/jun/10/nokia-mobile-phone.

